# LONELINESS AMONG OLDER IMMIGRANTS LIVING IN SUBSIDIZED SENIOR HOUSING: DOES PERCEIVED SOCIAL COHESION MATTER?

**DOI:** 10.1093/geroni/igac059.2926

**Published:** 2022-12-20

**Authors:** Jihye Baek, BoRin Kim, Sojung Park, Byeongju Ryu

**Affiliations:** Washington University in St. Louis, St. Louis, Missouri, United States; University of New Hampshire, Durham, New Hampshire, United States; Washington University in Saint Louis, Saint Louis, Missouri, United States; Washington University in St. Louis, St. Louis, Missouri, United States

## Abstract

The study compared the level of loneliness among older immigrants living in subsidized senior housing to non-immigrant residents. Also, the role of perceived social cohesion on loneliness was investigated, focusing on the differential influence among older immigrants and non-immigrants. Residents of subsidized senior housing in St. Louis and the Chicago area were recruited, and 182 responses were used in the analysis (126 immigrants and 56 non-immigrants). Descriptive analysis examined the difference in loneliness between immigrants and non-immigrants. Also, multiple regression models estimated the association between 1) immigrant status and loneliness, 2) perceived social cohesion and loneliness, and 3) the moderating role of immigrant status. Linear regression model showed that there was no significant difference between loneliness levels between immigrants and non-immigrants. However, perceived social cohesion was negatively associated with loneliness (β=-.338, SE= .025, p < .001). Additionally, immigration status moderated the relationship between perceived social cohesion and loneliness (β=-1.143, SE= .052, p < .01), which implies that immigrants may benefit more from higher perceived social cohesion in terms of loneliness compared to their non-immigrant counterparts. High perceived social cohesion might be an important community-level protective factor against loneliness in old age, especially for low-income older adults living in subsidized senior housing. Creating socially cohesive environments, particularly for this subgroup, would be crucial for mitigating loneliness and facilitating aging-in-place.

